# Medication profiles of patients undergoing dental treatment under general anesthesia or sedation in special needs dentistry

**DOI:** 10.3389/froh.2026.1889038

**Published:** 2026-07-09

**Authors:** Hitoshi Higuchi, Natsumi Naoi, Kaho Noda, Midori Inoue, Hitomi Ujita, Yukiko Nishioka, Saki Miyake, Takuya Miyawaki

**Affiliations:** 1 Department of Dental Anesthesiology, Okayama University Hospital, Okayama, Japan; 2 Department of Dental Anesthesiology and Special Care Dentistry, Okayama University Graduate School of Medicine, Dentistry and Pharmaceutical Sciences, Okayama, Japan; 3 Department of Dental Anesthesiology, Graduate School of Medical and Dental Sciences, Institute of Science Tokyo, Tokyo, Japan

**Keywords:** antiepileptic drugs, antipsychotics, dental anesthesia, general anesthesia, polypharmacy, sedation, special needs dentistry

## Abstract

**Introduction:**

Patients with intellectual disabilities, autism spectrum disorder, and related conditions often require sedation or general anesthesia to receive dental treatment. Many of these patients routinely take multiple medications, including antiepileptic and antipsychotic drugs, which may influence anesthetic management through drug interactions. However, medication use among patients receiving dental anesthesia in special needs dentistry has not been well investigated. This study aimed to clarify patterns of oral medication use in this population.

**Methods:**

This retrospective descriptive study included patients who underwent dental treatment under general anesthesia or intravenous sedation at Okayama University Hospital, Japan, between April 2022 and March 2025. Demographic characteristics, anesthesia-related variables, and regularly prescribed oral medications were collected from electronic medical records. Medications were categorized by pharmacological class, and descriptive statistical analyses were performed.

**Results:**

A total of 424 patients were analyzed. The mean age was 27.6 ± 15.3 years, and 67.4% were male. General anesthesia was used in 70.5% of patients. Overall, 71.0% regularly used oral medications, and polypharmacy, defined as the use of five or more medications, was observed in 23.5%. Antiepileptic drugs (36.8%) and antipsychotics (35.4%) were the most frequently prescribed medication classes. Risperidone and sodium valproate were the most commonly prescribed medications.

**Conclusion:**

Despite the relatively young age of the study population, medication use and polypharmacy were highly prevalent. The frequent use of antiepileptic and antipsychotic medications highlights the importance of understanding potential drug interactions during anesthetic management in special needs dentistry.

## Introduction

Among patients with intellectual disabilities, developmental disorders, mental illnesses, and related conditions, there are those who are unable to receive appropriate dental care despite a clear need for treatment. This is often due to fear or anxiety regarding dental treatment, or a lack of understanding, which may hinder their willingness to seek care. For these patients, behavioral management techniques using sedation or general anesthesia play a crucial role in the field of special needs dentistry (1). The appropriate application of these techniques can reduce anxiety and involuntary movements, enabling dental treatment to be performed safely and smoothly. Furthermore, these approaches not only alleviate pain and psychological distress for patients during treatment but also enhance the safety and reliability of procedures for dental professionals. However, the indication for anesthesia is often related to severe behavioral management difficulties, limited cooperation, or complex medical conditions, which may distinguish them from the broader special-needs population encountered in routine dental practice.

People with intellectual disabilities and developmental disorders have comorbid conditions and often take multiple medications on a daily basis to manage these conditions and the associated behavioral issues. Previous reports have shown that the use of antiepileptic drugs and antipsychotics is common among these patients (2, 3). Consequently, many patients in special needs dentistry who require dental treatment under general anesthesia or sedation are taking these medications, and healthcare professionals involved in special needs dentistry are frequently required to review complex medication regimens. Therefore, understanding the prevalence and patterns of medication use in this clinical setting may help improve perioperative planning and risk assessment for dental treatments that require special consideration.

However, information regarding these medications among patients actually undergoing dental treatment under anesthesia in special needs dentistry is limited, and the actual situation remains unclear. Therefore, the objective of this study is to clarify the prevalence and patterns of regular medication use among patients undergoing dental treatment under general anesthesia or intravenous sedation in special needs dentistry.

## Materials and methods

### Study design and ethical approval

This retrospective descriptive study was conducted at Okayama University Hospital. The study protocol was reviewed and approved by the Ethics Committee of the Okayama University Graduate School of Medicine, Dentistry, and Pharmaceutical Sciences and Okayama University Hospital (approval number: 2506–025; approval date: June 20, 2025).

The requirement for written informed consent was waived in accordance with the Japanese Ethical Guidelines for Life Science and Medical Research Involving Human Subjects because of the retrospective design and the use of anonymized clinical data. All procedures were conducted in accordance with the principles of the Declaration of Helsinki.

Because this study did not involve prospective enrollment or intervention, it was not registered in a clinical trial registry.

### Study setting and participants

This study was conducted in the Department of Dental Anesthesiology at Okayama University Hospital. The study included patients who required dental treatment under general anesthesia and/or intravenous sedation at the Special Needs Dentistry Center of Okayama University Hospital, a tertiary care facility in Japan that provides dental care for patients with special medical needs, between April 2022 and March 2025.

The study population consisted of patients with intellectual disabilities, autism spectrum disorder, or other medical or behavioral conditions that made conventional dental treatment difficult. The decision to administer anesthesia was made by the treating dentist based on a comprehensive clinical evaluation, including the patient's level of cooperation, severity of the dental condition, and anticipated procedural complexity.

All consecutive patients who underwent anesthesia management for dental treatment during the study period were included. No additional inclusion criteria were applied in order to minimize selection bias and to reflect real-world clinical practice. Patients were excluded only if relevant clinical data were incomplete or unavailable in the medical records.

### Data collection

Patient demographic characteristics and anesthesia-related variables were retrospectively collected from electronic medical records and anesthesia records. Data were extracted using standardized data collection forms, and original source documents were reviewed when necessary to ensure accuracy.

The primary outcome of this study was the pattern of regular medication prescribed by patients' primary care physicians among patients undergoing anesthesia management. Medication data were obtained from the patients' medication notebooks at the time of preoperative assessment. Over-the-counter medications and dietary supplements were excluded from this analysis. Based on official drug labels and pharmacological classifications, regular medications were categorized into the following 15 groups: antiepileptic drugs, antipsychotics, antidepressants and anxiolytics, hypnotics, cardiovascular drugs, antidiabetic agents, antiplatelet agents, anticoagulants, hormonal medications, antiallergic drugs, gastric medications, intestinal regulators and laxatives, lipid-lowering agents, anti-Parkinson drugs, and others.

Secondary variables included age, sex, type of disability, anesthesia method (general anesthesia or sedation), whether patients used oral medications, number of oral medications, and detailed information on oral medications.

For patients who underwent anesthesia multiple times during the study period, only the most recent episode was included in the analysis to avoid duplication and potential bias related to repeated measurements. In cases where both general anesthesia and sedation were used during the study period, the patient was classified into the general anesthesia group for analysis. Polypharmacy was defined as the concurrent regular use of five or more medications.

### Data management

All collected data were anonymized prior to analysis. Data were stored securely and handled in accordance with institutional data protection policies. Only authorized investigators had access to the study data.

### Data analysis

This study was conducted as a descriptive epidemiological study. Continuous variables were summarized using descriptive statistics, such as means, standard deviations, and ranges, as appropriate. Categorical variables were presented as frequencies and percentages.

No inferential statistical analyses were performed because the primary objective of this study was to describe the characteristics of the study population and patterns of medication use rather than to test specific hypotheses or evaluate associations.

## Results

Between April 2022 and March 2025, a total of 425 patients underwent dental treatment under general anesthesia or intravenous sedation, accounting for 825 anesthetic management procedures. One patient was excluded due to insufficient data regarding the primary outcome measures. Consequently, 424 patients were included in the final analysis.

The demographic, clinical characteristics, and anesthetic management of the study population are summarized in Table 1. The mean age of the participants was 27.6 years. Although the majority of patients were young adults, the study also included elderly individuals aged over 70 years. There were more male than female patients, and the most common disabilities were intellectual disabilities and autism spectrum disorder. In addition, approximately 300 patients underwent dental treatment under general anesthesia.

**Table 1:** Demographic, clinical characteristics and anesthetic management of the subject cases.

Total number (*n*)	424
Age (years)*	27.6 ± 15.3 (5–73)
Age group(*n*)	
≤ 9	56 (13.2%)
10–19	84 (19.8%)
20–29	117 (27.6%)
30–39	69 (16.3%)
40–49	54 (12.7%)
50–59	32 (7.5%)
60–69	9 (2.1%)
≥ 70	3 (0.7%)
Gender (Male/Female) (*n*)	286 (67.5%)/138 (32.5%)
Major disability type (*n*)**	
Intellectual disability	317
Autism spectrum disorder	201
Cerebral palsy	29
Down syndrome	21
Anesthesia method (*n*)***	
General Anesthesia	299 (70.5%)
Intravenous sedation	125 (29.5%)

* Mean ± SD (Min- Max).

** Duplicates included.

*** Patients who underwent both sedation and general anesthesia were classified as general anesthesia.

The usage patterns of oral medications in the study population are shown in Table 2. More than 70% of patients were taking medication regularly, and 23.5% were prescribed five or more types of medication, suggesting that polypharmacy was widespread in this population. By drug type, antiepileptic drugs and antipsychotics were the most common, each accounting for more than 35% of the total. In addition, 77 patients were taking both antiepileptic drugs and antipsychotic drugs, accounting for 18.2% of all participants and 25.6% of those taking one or more types of medication. This reflects the highly specific medical characteristics of dental patients requiring special care.

**Table 2:** Oral medication profile of the subject cases.

Presence of oral medications (Yes)*	301 (71.0%)		
Number of oral medications	Number (*n*)	Percentage (%)	
		Among all participants	Among oral medication group*
1–4	202	47.6	67.1
5–9	85	20.2	28.2
Over 10	14	3.3	4.7
Classification			
Antiepileptic drugs	155	36.6	51.5
Antipsychotics	150	35.4	49.8
Probiotics/Laxatives	91	21.5	30.2
Antiallergic drugs	76	17.9	25.2
Hypnotics	57	13.4	17.2
Antidepressants/Anxiolytics	39	9.2	13.0
Gastrointestinal drugs	30	7.0	10.0
Antihypertensive drugs	24	5.7	8.0
Antiparkinsonian drugs	23	5.4	7.6
Antihyperlipidemic drugs	16	3.8	5.3
Hormonal agents	12	2.8	4.0
Antidiabetic drugs	6	1.4	2.0
Anticoagulants	4	0.9	1.3
Antiplatelet drugs	3	0.7	1.0

* Participants taking at least one medication.

Table 3 shows the top 10 most frequently prescribed medications. The most commonly prescribed medication was risperidone, followed by sodium valproate. Both of these medications were used in more than 20% of patients, suggesting that medications commonly used to treat neurological and psychiatric disorders were prescribed at notably high frequencies in this population.

**Table 3:** Top 10 oral medications.

Drug name	Classification	Number(n)	Percentage (%)	
			Among all participants	Among oral medication group*
Risperidone	Antipsychotics	100	23.6	33.2
Sodium valproate	Antiepileptic drugs	95	22.4	31.6
Magnesium Oxide	Laxative	46	10.8	15.3
Carbamazepine	Antiepileptic drugs	42	9.9	14.0
Aripiprazole	Antipsychotics	41	9.7	13.6
Levomepromazine maleate	Antipsychotics	29	6.8	9.6
Levetiracetam	Antiepileptic drugs	25	5.9	8.3
Butyrate-producing bacteria	Probiotics	18	4.2	6.0
Quetiapine fumarate	Antipsychotics	16	3.8	5.3
Lemborexant	Hypnotics	16	3.8	5.3

* Participants taking at least one medication.

## Discussion

This study revealed that, in the field of special needs dentistry, as many as 70% of patients undergoing anesthesia were taking at least one medication. Furthermore, among these patients, 50% were taking either antiepileptic drugs (AEDs) or antipsychotic medications. More specifically, sodium valproate as an AED and risperidone as an antipsychotic drug each accounted for over 30% of prescriptions.

Medication use is highly prevalent in the general adult population. Epidemiological data from the Centers for Disease Control and Prevention indicate that approximately 64.8% of adults in the United States reported using at least one prescription medication within the past year (4). In addition, recent large-scale data suggest that 46.3% of adults used at least one prescription medication within the past 7 days, further supporting the widespread nature of medication use in the general population (5). Medication use is known to increase with age. Several studies have demonstrated that more than 80% of adults aged 65 years and older take at least one medication (4, 6), and polypharmacy—commonly defined as the concurrent use of five or more medications—is observed in approximately 40% of this population (6–9). These findings suggest that a high burden of medication use is typically associated with older age groups.

In contrast, the patients included in the present study were relatively young, with a mean age of 27.6 years. Despite this, 70% of patients undergoing anesthesia in the field of special needs dentistry were taking medications. Furthermore, the prevalence of polypharmacy in this study was 23.5%. In the aforementioned epidemiological survey, the prevalence of prescription medication use among individuals aged 18–44 years was 48.4% (4). Similarly, Kantor et al. reported that the prevalence among those aged 20–39 years was 35%, with a polypharmacy rate of 3.1% (6). Therefore, the present results indicate that patients with special needs exhibit a markedly elevated burden of medication use even at a younger age, highlighting their distinct clinical characteristics.

The high prevalence of prescribed medications and the tendency toward polypharmacy observed in this study support the notion that patients attending special needs dental services commonly have underlying medical conditions. In particular, the markedly high use of AEDs and antipsychotics suggests that the medical background of this population is not uniform but rather characterized by a specific pattern of comorbidities.

In the present study, AEDs were the most frequently prescribed class of medications, which may reflect a relatively high prevalence of epilepsy in this population. The prevalence of epilepsy in the general population has been reported to be approximately 0.6% (10, 11), and the use of AEDs in the general population is typically limited to a few percent (6). In contrast, epilepsy is substantially more common among individuals with intellectual disabilities, with a pooled prevalence of 22.2% (95% CI: 19.6–25.1%) reported in a meta-analysis of 38 studies (12). Furthermore, the prevalence of epilepsy has been shown to increase with the severity of intellectual disability (12, 13).

Among the AEDs, valproate was the most frequently prescribed agent. Valproate is a broad-spectrum AED with well-established efficacy against generalized seizures, and several randomized controlled trials have demonstrated its superior seizure control compared with other agents (14, 15). In recent years, concerns regarding teratogenicity and adverse neurodevelopmental outcomes have led to significant restrictions on its use in women of childbearing potential. Nevertheless, in patients for whom these safety concerns are less relevant, valproate remains one of the most effective therapeutic options (16–18). Therefore, its high utilization in this study may reflect the clinical need for effective seizure control in a population with a high prevalence of severe neurological conditions.

The high rate of antipsychotic use observed in individuals with intellectual disability in this study may reflect the use of these medications for “challenging behaviour” or behavioural disturbances. A large UK primary care cohort study reported that the rate of psychotropic prescribing in people with intellectual disability exceeded the recorded prevalence of mental illness, and that antipsychotics were frequently prescribed in individuals without a diagnosis of severe mental illness (19). These findings suggest that antipsychotics are used not only for clearly defined psychiatric conditions such as schizophrenia or bipolar disorder, but also for behavioural problems including agitation, aggression, self-injury, and severe irritability.

However, although antipsychotic use for challenging behaviour is widespread, the evidence regarding its efficacy and safety remains limited, with inconsistent findings across studies (20). Accordingly, a guideline recommends psychological and environmental interventions as first-line treatments, and suggests that pharmacological interventions should be reserved for cases with severe risk or when non-pharmacological approaches have failed (21). Taken together, these findings suggest that individuals included in the present study may have had a certain degree of behavioural difficulties.

Among pharmacological treatments, risperidone and aripiprazole have relatively more evidence supporting their use for challenging behaviour. Both agents have demonstrated efficacy in reducing irritability and aggression associated with autism spectrum disorder, and head-to-head trials have not shown significant differences in efficacy between the two drugs (22, 23). Therefore, these medications are generally considered to have comparable clinical effectiveness.

Despite this, risperidone was used at a markedly higher rate in the present study, a pattern that is also reported in other countries (24, 25). One possible explanation is that risperidone was introduced earlier into clinical practice and has accumulated a larger body of evidence, including randomized controlled trials and long-term observational studies. In addition, its pharmacological profile, characterized by dopamine D2 and serotonin 5-HT2A receptor antagonism, may result in behavioural calming effects that are readily recognized in clinical settings, particularly in the management of agitation and aggression. Furthermore, practical factors such as the availability of multiple formulations, ease of dose adjustment, and extensive clinical experience may also contribute to its preferential use.

The high prevalence of antiepileptic drugs and antipsychotics observed in the present study is generally consistent with previous reports involving individuals with intellectual disabilities and developmental disorders. However, the prevalence of antiepileptic drug use in our study (36.8%) was notably higher than the reported prevalence of epilepsy among individuals with intellectual disabilities. Furthermore, it is well known that polypharmacy is common among people with intellectual or developmental disabilities, and the prevalence of polypharmacy in this population also increases with age, just as it does in the general population (26). Since the study population consisted primarily of young adults, and a report indicated that the rate of polypharmacy in a relatively similar group (aged 18–39) was 13.3%, our study results indicate a relatively high prevalence of it (27). These findings do not necessarily indicate that the medications prescribed to patients undergoing anesthesia management were inappropriate; rather, they suggest that these patients constitute a subgroup characterized by clinical complexity, a greater burden of neurological and behavioral comorbidities, and potentially more severe functional impairments. Because the indication for anesthesia-assisted dental treatment is often related to severe behavioral management difficulties, limited cooperation, or complex medical conditions, the present study might represent the more clinically challenging end of the special-needs spectrum.

Although this study did not evaluate the effects of these medications on anesthesia-related outcomes, it is clinically very important to understand this possibility, as these medications may influence anesthesia management. AEDs and antipsychotic drugs, which were found to be particularly common in this study, have both been reported to interact with anesthetic agents. Valproic acid, which was frequently used in this survey, has been reported to potentiate the effects of the intravenous anesthetic propofol through mechanisms such as enhanced central nervous system depression and competition for protein binding sites (28–30). Furthermore, AEDs are known to induce or inhibit the metabolic enzyme cytochrome P450 (CYP) (31), thereby affecting drug metabolism. In particular, carbamazepine and phenytoin induce CYP3A4 and accelerate the metabolism of midazolam, a benzodiazepine (32, 33). Thus, the use of AEDs may potentiate or attenuate the effects of various anesthetics, and it is important to be aware of this when managing anesthesia.

On the other hand, antipsychotic drugs possess *α*1-receptor blocking effects (34); when epinephrine is administered, β2-receptor activity becomes relatively dominant, leading to what is known as an epinephrine reversal, which may result in a decrease in blood pressure (35). However, the receptor binding profiles of antipsychotic drugs are diverse (36). While risperidone, which was used very frequently in this study, has a relatively strong *α*1-blocking effect, aripiprazole, which was the second most frequently used drug after risperidone, is considered to have a somewhat weaker effect (37, 38). When using adrenaline-containing local anesthetics or catecholamine preparations in these patients, close attention must be paid to changes in hemodynamics.

There are several limitations to consider when interpreting the results of this study. First, as this is a retrospective observational study relying on data collected from medical records, the findings may be subject to the influence of incomplete documentation or missing information. Second, this study focused on patients with special needs at a single university hospital; consequently, this study may have a higher proportion of patients with severe intellectual or developmental disabilities compared to general dental practices. Consequently, the prevalence of antiepileptic and antipsychotic medication use may be overestimated compared to the general population, and caution is required when generalizing the results. Furthermore, this study reflects prescribing practices in Japan, and the results may have been influenced by Japanese clinical guidelines in effect at the time. In addition, because this study did not include a comparison group of individuals with special healthcare needs who did not undergo anesthesia-assisted dental treatment, the extent to which the observed medication patterns are specific to patients requiring anesthesia-supported care remains uncertain. Third, while this study investigated the use of oral medications among patients who underwent anesthesia management, it did not evaluate the clinical impact of these medications on anesthesia management. Consequently, it is unclear to what extent interactions between antiepileptic and antipsychotic drugs and anesthesia-related medications affect clinical outcomes. Furthermore, this study did not evaluate medication dosage, duration of therapy, adherence, serum drug concentrations, or the continuation and discontinuation of medications during the perioperative period. Investigating these factors requires a more rigorous study design, including detailed information on medication use, anesthesia techniques, and clinical outcomes. Therefore, future prospective studies incorporating anesthesia-related complications and postoperative outcomes are warranted.

In conclusion, we investigated the medication use of patients requiring anesthetic management in the field of special needs dentistry. The results showed that, despite the relatively young age of the study patients, approximately 70% required regular medication, and polypharmacy (the use of five or more medications) was observed in about 23%. In particular, antiepileptic and antipsychotic medications were frequently prescribed. These findings provide practical information regarding the medication profiles frequently encountered during anesthesia management in the clinical setting of special needs dentistry.

## Data Availability

The raw data supporting the conclusions of this article will be made available by the authors, without undue reservation.
